# East Coast Fever Caused by *Theileria parva* Is Characterized by Macrophage Activation Associated with Vasculitis and Respiratory Failure

**DOI:** 10.1371/journal.pone.0156004

**Published:** 2016-05-19

**Authors:** Lindsay M. Fry, David A. Schneider, Charles W. Frevert, Danielle D. Nelson, W. Ivan Morrison, Donald P. Knowles

**Affiliations:** 1 Animal Disease Research Unit, Agricultural Research Service, US Department of Agriculture, Pullman, Washington, United States of America; 2 Department of Veterinary Microbiology & Pathology, Washington State University, Pullman, Washington, United States of America; 3 The Roslin Institute, University of Edinburgh, Easter Bush, Midlothian, United Kingdom; 4 Department of Comparative Medicine Center of Lung Biology, University of Washington School of Medicine, Seattle, Washington, United States of America; Leibniz Institute for Natural Products Research and Infection Biology- Hans Knoell Institute, GERMANY

## Abstract

Respiratory failure and death in East Coast Fever (ECF), a clinical syndrome of African cattle caused by the apicomplexan parasite *Theileria parva*, has historically been attributed to pulmonary infiltration by infected lymphocytes. However, immunohistochemical staining of tissue from *T*. *parva* infected cattle revealed large numbers of CD3- and CD20-negative intralesional mononuclear cells. Due to this finding, we hypothesized that macrophages play an important role in *Theileria parva* disease pathogenesis. Data presented here demonstrates that terminal ECF in both Holstein and Boran cattle is largely due to multisystemic histiocytic responses and resultant tissue damage. Furthermore, the combination of these histologic changes with the clinical findings, including lymphadenopathy, prolonged pyrexia, multi-lineage leukopenia, and thrombocytopenia is consistent with macrophage activation syndrome. All animals that succumbed to infection exhibited lymphohistiocytic vasculitis of small to medium caliber blood and lymphatic vessels. In pulmonary, lymphoid, splenic and hepatic tissues from Holstein cattle, the majority of intralesional macrophages were positive for CD163, and often expressed large amounts of IL-17. These data define a terminal ECF pathogenesis in which parasite-driven lymphoproliferation leads to secondary systemic macrophage activation syndrome, mononuclear vasculitis, pulmonary edema, respiratory failure and death. The accompanying macrophage phenotype defined by CD163 and IL-17 is presented in the context of this pathogenesis.

## Introduction

*Theileria parva* is an intracellular apicomplexan parasite of Cape buffalo (*Syncerus caffer*) and *Bos indicus* and *Bos taurus* cattle in sub-Saharan Africa. Mortality rates are high in most imported *Bos taurus* breeds and indigenous breeds raised in non-endemic areas [1]. *T*. *parva* kills over one million cattle each year in sub-Saharan Africa, resulting in severe economic disadvantage for pastoral farmers. Like the closely related protozoan pathogens *Plasmodium* and *Babesia*, and the distantly related protozoan pathogens *Trypanosoma* and *Leishmania*, which cause disease in animals and humans, *T*. *parva* is arthropod-borne.

*T*. *parva* is primarily transmitted by the three-host tick *Rhipicephalus appendiculatus*. Following transmission to cattle by infected ticks, sporozoites enter lymphocytes and mature into multinucleated schizonts. Schizonts induce constitutive activation of multiple host-cell signal transduction pathways, preventing apoptosis and causing cell transformation and marked lymphoproliferation [2–5]. Association of the schizont with the host cell mitotic spindle apparatus allows the schizont to divide in concert with infected host cells, and also minimizes schizont exposure to extracellular clearance mechanisms. Prior to data presented here, clinical disease, including terminal ECF, was thought to be driven solely by lymphoproliferation and resultant organ infiltration by infected, transformed lymphocytes.

Clinical disease (East Coast Fever) is characterized by marked peripheral lymphadenopathy, fever, anorexia and respiratory distress. Small numbers of infected cattle develop neurologic signs (turning sickness), due to blockage of cerebral vessels by clusters of parasitized cells. Clinicopathologic changes described in end-stage disease include severe leukopenia, thrombocytopenia, lymphocytolysis in lymphoid tissues and hemorrhage [6, 7]. The causes of leukopenia, which involves both lymphoid and myeloid cells, are unclear but likely include rupture of infected lymphocytes during merozoite production, and lysis of uninfected cells in lymphoid tissues. Treatment with antiparasitic drugs, such as buparvaquone early in the course of infection, can lessen disease severity, but does not always eliminate the parasite [8]. Immunization of cattle by concurrent infection of cattle with live *T*. *parva* sporozoites and treatment with long-acting oxytetracycline, known as the infection and treatment method (ITM) [9], results in transient clinical reactions, apparently due to the impact of oxytetracycline on parasite replication. At least one *T*. *parva* strain included in the ITM cocktail results in long-term *T*. *parva* infection [10].

Cattle that survive natural infections or are immunized using ITM develop solid immunity to similar strains. In these animals, the protective immune response is largely mediated by major histocompatibility complex (MHC) class I-restricted CD8^+^ cytotoxic T lymphocytes (CTL) specific for *T*. *parva* schizont-infected lymphocytes [11] and is often strain-specific [12]. Cattle immunized using ITM also generate MHC class II-restricted CD4^+^ T cells specific for *T*. *parva* schizont-infected lymphocytes [12, 13]. Like parasite-specific CTLs, parasite-specific helper T cells are sometimes strain-specific [12].

Unfortunately, animals infected with *T*. *parva*, even with concurrent treatment, often fail to control the parasite and succumb to severe ECF within three weeks of infection. Severely affected cattle usually succumb to pulmonary edema [6]. Widespread tissue infiltration by transformed lymphocytes, including infiltration of alveolar septae, has been documented by histopathologic evaluation [6, 14–16]. These data led to the understanding that pulmonary infiltration by transformed lymphocytes was associated with respiratory failure; however, past histologic evaluation of affected tissues was limited.

As part of vaccine development efforts, infection and treatment experiments were initiated to study development of immunity to *T*. *parva*. Despite intensive supportive care, a subset of animals developed severe ECF intractable to treatment. To assure a complete understanding of the disease pathogenesis, these animals were fully evaluated by necropsy, histopathology and follow-up immunohistochemical (IHC) studies.

Two IHC studies on tissue from infected cattle have been published [17, 18], and support previous histologic data that large numbers of T cells infiltrate the lungs of cattle with ECF. While our initial IHC analyses also demonstrate abundant T cells within the lungs of cattle, we also documented a large population of CD3- and CD20-negative mononuclear cells within tissues of affected cattle. Based on these studies, we hypothesized that histiocytic cells (cells of the monocyte/macrophage lineage) play a role in the development of acute ECF in cattle. Histology, IHC, and morphometry were used to garner an understanding of the role macrophages play in the development of acute ECF.

Although some of the components of the immune response to *T*. *parva* are well-understood, the pathogenesis of lethal disease, including the possible role of aberrant immune responses, was hitherto largely unexplored. Since macrophages can serve both immunostimulatory and immunosuppressive roles, and thus alter the efficacy and character of the adaptive immune response, we also sought to provide initial characterization of the histiocytic response in *T*. *parva*. CD163 is a macrophage scavenger receptor that is generally up-regulated in anti-inflammatory, immunosuppressive states, and also in response to severe tissue injury ([19, 20]). In contrast, IL-17 is a pro-inflammatory cytokine produced by many leukocytes (including macrophages) that is often associated with severe immunopathology ([21–23]). We used immunohistochemical labeling of CD163 and IL-17 to provide basic phenotypic information about the macrophage response in *T*. *parva*. These data were coupled with clinical data to generate a more detailed understanding of the immunopathogenesis of acute ECF.

Data presented here expand ECF pathogenesis to include a macrophage-centric inflammatory response. While a robust innate immune response is likely required to trigger a disease-limiting adaptive immune response to *T*. *parva*, excessive macrophage activation and resultant tissue destruction likely trigger ECF. Importantly, infection with lethal doses of *T*. *parva* was found to cause severe lymphohistiocytic vasculitis of the lungs, lymph nodes, spleen and liver, and these organs were shown to contain large numbers of CD163^+^ and IL-17^+^ macrophages. We propose that pulmonary edema and respiratory failure during ECF are due to the development of pulmonary vasculitis, and that the induction of a multisystemic histiocytic response contributes significantly to clinical disease in ECF.

## Materials and Methods

### Holstein cattle (*Bos taurus*)

All animal experiments were approved by the Washington State University and University of Idaho Institutional Animal Care and Use Committees, protocol numbers 2013–66 (U of I) and 04515–002 (WSU). All therapeutic drugs were administered according to the manufacturer’s dosing instructions. This study utilized eight, three month-old Holstein steers from dairies in northern Idaho and central Washington. These cattle were acquired for initiation of vaccine development studies. Cattle were quarantined at the USDA-ARS-ADRU vector disease research barns for two weeks before the onset of the study, and received regular health checks from a licensed veterinarian at this time. Pre-infection complete blood counts (CBCs) and serum chemistry panels were normal. After quarantine, calves were housed in pairs in box stalls in the USDA-ARS-ADRU vector disease research barn. Stalls were bedded with wood shavings. Animals had continuous access to fresh water and were fed grass and alfalfa hay and a small amount of Calf Manna (MannaPro®, USA) twice daily. Mechanical heating and cooling units, coupled with open-air ventilation were used to maintain the barn at approximately 16°C. Calves were exposed to overhead lighting from 7 a.m. to 5 p.m. daily, and were closely monitored by animal care staff and veterinarians.

Five animals were infected with *T*. *parva* via subcutaneous injection of 0.2–0.5 mL of cryopreserved *T*. *parva* Muguga sporozoite stabilate Ed80 in the left parotid region. Three uninfected animals were maintained as negative controls. Following infection, complete physical examination, including rectal temperature, palpation of peripheral lymph nodes and thoracic auscultation, was performed on each animal at least once per day. At the onset of pyrexia (rectal temperature ≥ 39.4°C), CBCs were performed regularly to monitor leukocyte, erythrocyte, and platelet counts. As soon as peripheral lymph node enlargement was detected, needle aspirates were collected from affected nodes once daily, and Giemsa-stained smears of aspirates examined for schizont-infected lymphocytes.

Two animals were co-treated with Liquamycin® (Zoetis, USA), a long-acting form of oxytetracycline (LA OTC) at the time of infection. In the three remaining calves, Terramycin® (Zoetis, USA), a short-acting oxytetracycline (SA OTC) was administered intramuscularly every 24 hours after the onset of pyrexia in an attempt to curtail schizont parasitemia. In all calves, pyrexia was controlled via parenteral administration of flunixin meglumine (Pfizer Animal Health, USA), and anorexic calves were given Resorb® (Zoetis, USA) oral electrolyte solution three times a day. If animals developed dyspnea or tachypnea, furosemide (Bayer Healthcare, USA) was administered via intramuscular injection to decrease pulmonary edema. Buparvaquone (Bimeda, Ireland) was administered via intramuscular injection every 48 hours to aid in controlling disease in two calves.

Three inoculated calves developed severe dyspnea that failed to respond to the aforementioned treatment protocols and were euthanized via intravenous injection of Fatal Plus (Vortech Pharmaceuticals, USA), and one calf died suddenly of respiratory failure just before euthanasia could be administered. In each case, a complete necropsy was performed within twelve hours of death. Table 1 summarizes information on each infected Holstein calf included in this study.

**Table 1:** Animal Identification, Infection, Treatment and Outcome.

Animal Number	Stabilate Dose (mL)	Antibiotic Treatment	Buparvaquone	Outcome
1412	0.5	LA OTC, ITM	Yes	Survived
1415	0.3	LA OTC, ITM	Yes	Euthanized
1419	0.2	SA OTC	No	Euthanized
1420	0.2	SA OTC	No	Died
1435	0.2	SA OTC	No	Euthanized

### Histopathology and immunohistochemistry, Holstein cattle

Tissue sections were fixed in 10% neutral buffered formalin for at least seven days. For basic histopathology, formalin-fixed sections of lung, lymph node, heart, liver, spleen, kidney, adrenal gland, forestomachs, intestines, brain and pituitary gland were trimmed, routinely processed, stained with hematoxylin and eosin (H&E), and examined with a light microscope. Immunophenotyping of the leukocyte infiltrates was performed using immunohistochemical (IHC) detection of CD3 (T cell marker [24]), CD20 (B cell marker [25]), IBA-1 (pan-histiocytic (monocyte/macrophage/dendritic cell) marker [26, 27]), CD163 (macrophage marker [28]), and, due to recent studies on its importance in lesion development in several protozoal infections, interleukin-17 [21–23, 29]. Detection of *T*. *parva* schizonts was performed using a monoclonal antibody to the *T*. *parva* schizont surface-bound polymorphic immunodominant molecule (PIM) antigen [18, 30]. The species of origin, isotype, source, concentration, and Antibody Registry numbers (where applicable) of the primary antibodies used in this study are provided in Table 2.

**Table 2:** Antibodies used for IHC and Fluorescent Co-Labeling IHC.

Target	Name	Species and Isotype	Concentration (μg/mL)	Source	Antibody Registry ID
CD3	A0452	Rabbit, polyclonal	6	Dako	AB_2335677
CD20	Rb9013	Rabbit, polyclonal	0.13	Neomarkers	AB_149765
CD163	MCA1853	Mouse, IgG1	2.5	Serotec	AB_2074540
IBA-1	IBA-1	Rabbit, polyclonal	2	Wako	AB_839504
IL-17	Ab79056	Rabbit, IgG	10	AbCam	AB_1603584
PIM	ILS-40	Mouse, IgG	3.1	Ivan Morrison	

For all IHC, 2–3 μm thick serial sections of formalin-fixed, paraffin-embedded tissues were placed onto positively charged glass slides (Superfrost®/Plus, Fisher Scientific, Pittsburg, PA). An automated processor (Discovery XT, Ventana Medical Systems,Tucson, AZ) was used for deparaffinization, antigen retrieval and immunolabeling. For single immunolabeling, sections were incubated with the primary antibody for two hours, followed by the Ventana universal secondary antibody and detection with the Discovery DAB® (3,3’–diaminobenzadine chromagen) Map Kit (Ventana). For double-labeling studies, sections were then incubated with the second primary antibody for three hours, followed by the Ventana universal secondary antibody and detection with the Discovery® RedMap (Red/Napthol chromagen) Kit (Ventana). All slides were then counterstained with hematoxylin, and evaluated using a light microscope. Immunostaining of tissues from an uninfected calf, as well as incubation of tissues with irrelevant, isotype-matched primary antibodies or substitution of primary and secondary antibodies with antibody diluent were used as controls in all reactions.

### Fluorescent Immunohistochemical Co-Labeling

Thin section deparaffinization, antigen retrieval and fluorescence immunohistochemistry were performed using the automated slide staining platform, Discovery XT (Ventana Medical Systems). Following extended deparaffinization (75°C), epitope retrieval was achieved using a tris-based buffer, pH 8.5, (Cell Conditioning Solution CC1, Ventana Medical Systems) held at 95°C for ~60 minutes (“standard cell conditioning” protocol). Thereafter, the sequence of automated protocol steps for dual immunofluorescence labeling were: antibody blocking, application of 1^st^ primary antibody and detection steps using tyramide-FITC signal amplification (RUO anti-Rabbit or anti-Mouse IgG FITC, Ventana Medical Systems), denaturation (32 minutes at 95°C), application of 2^nd^ primary antibody and detection steps using Goat anti-Rabbit or anti-Mouse IgG conjugated to AlexaFluor 647 (Invitrogen).

Dual labeling was performed to detect cells co-expressing IL-17 (1^st^ primary antibody, 4 hours incubation, Table 2) and each of the cell lineage markers CD3, CD163, and IBA-1 (2^nd^ primary antibodies, 12 hours incubation, Table 2). Control reactions included substitution of the 1^st^ primary with 10 μg/mL Rabbit non-immune serum (Dako), the 2^nd^ primary with 6 μg/mL Rabbit non-immune serum or 2.5 μg/mL of a Mouse monoclonal IgG1 irrelevant epitope antibody. No fluorescence bleed through or cross-reactivity were observed in these control reactions.

Dual labeling of CD163 and IBA-1 was conducted as above for IBA-1 (2 μg/mL) as the 2^nd^ primary antibody, but substituting MCA1853 (1 μg/mL, 2 hour incubation) as the 1^st^ primary antibody. No fluorescence bleed through or cross-reactivity were observed when substituting 1 μg/mL of a Mouse monoclonal IgG1 irrelevant epitope antibody as the 1^st^ primary antibody or 2 μg/mL Rabbit non-immune serum as the 2^nd^ primary antibody.

Epifluorescence microscopy was conducted using an Axio Imager.M1 microscope (Carl Zeiss Microimaging, Thornwood, NY) equipped with an X-Cite 120 Fl Illuminating system (EXFO Photonic Solutions, Mississauga, Ontario, Canada), an AxioCam MRm digital camera, and a computer workstation running AxioVision 4.8.1 imaging software. Fluorescence images were captured as a z-stack (step increment 275 nm) using an EC Plan-Neofluar 40x/1.3 oil M27 objective. Maximum intensity projections of z-stack images were processed using Fiji (an ImageJ-based open source image processing package) and figures organized using Photoshop Elements software.

### Boran Cattle (*Bos indicus*)

H&E stained sections of the left pre-scapular lymph nodes from twenty African Boran calves were evaluated. All cattle were used in an ECF infection kinetics study at the International Livestock Research Institute in Nairobi, Kenya in 1981 [31]. In the study, 17/20 cattle were infected with a lethal dose of *T*. *parva* Muguga, and three calves were used as negative controls. Following infection, pairs of cattle were euthanized on days 3, 6, 8, 10, 12, 14, and 16 days post infection. Of the remaining three infected animals, one was euthanized on day 18, one on day 19, and one animal succumbed to ECF on day 19. The negative control animals were euthanized at the end of the experiment. Lymph nodes were collected during postmortem examination, fixed in 10% neutral buffered formalin, and routinely processed for histopathology. Slides were stored until 2014 by Dr. Ivan Morrison, and were shipped to Pullman, WA for histologic evaluation.

### Morphometric Measurements of Lungs

To provide quantitative measurements of CD163, IBA-1 and IL-17 positive staining in lung tissue, whole slide digital images were collected and automated image analysis performed. Briefly, sections of lung from infected and control animals immunohistochemically labelled for IBA-1, CD163, and IL-17 as described above were scanned in bright field with a 20X objective using a Nanozoomer Digital Pathology slide scanner (Hamamatsu; Bridgewater, New Jersey). The whole slide digital images were then imported into Visiopharm software (Hoersholm, Denmark) for analysis. The Visiopharm Image Analysis module automatically detected tissue on slides, from which sections of lung were selected. For each marker, 184–234 mm^2^ of control calf lung tissue was analyzed and 497–625 mm^2^ of infected calf lung tissue was analyzed. 100% of each lung section was analyzed. Digital images were converted to gray scale values using RGB-R and RGB-B filters with a mean size of 5 pixels by 5 pixels. Visiopharm was trained to label positively stained cells for CD163, IBA-1 and IL17, and to detect background tissue counter stain (Hematoxylin) using an analyte-specific configuration based on a threshold of pixel values. Images were processed in batch mode using the configuration developed for each analyte to generate the desired outputs. For each analyte, the following were calculated: A. Area of positive immunolabeling, B. Area of hematoxylin stained tissue, C. Total area of tissue (A+B), and D. Ratio of positive analyte staining to total tissue area (A/C). Any non-specific staining (e.g., staining along the edge of tissues) on tissue sections or any tissue that showed substantial damage from processing was excluded from analysis. Based on output for each section analyzed, the mean value for each analyte was calculated by averaging the results obtained from each animal and then averaging the results for the control cattle and cattle infected with *T*. *parva*. Mean values for control and infected groups were compared using a one-tailed student’s t-test (α<0.05).

## Results

### Clinical disease kinetics of ECF in Holstein calves

Left parotid lymphadenopathy was noted 4–7 days post infection, and enlargement of contralateral and other regional lymph nodes developed by day 9. Schizonts were detected in Giemsa-stained aspirates of the left parotid lymph node within 24 hours of the development of lymphadenopathy, and the percentage of schizont-positive, blasting lymphocytes ranged from 9%-34% during acute disease. Large numbers of uninfected lymphoblasts were also detected in all aspirates. All cattle exhibited severe, sustained pyrexia (39.5–41.6°C) from 6–9 days post infection onward. Resting respiratory rates in all calves began to increase between days 6 and 11 post-infection, and cattle exhibited terminal dyspnea characterized by neck extension, rapid, shallow inspiration and marked expiratory effort consistent with restrictive pulmonary disease. During this time, crackles were auscultated over all lung fields. Between days 10 and 15, four of five animals developed petechiae over the gingival and scleral mucosae. Over the course of acute disease, the body weight of all animals decreased 25%-33%, and calves exhibited decreased skeletal muscle mass and increased skeletal prominence. Similar clinical reactions were observed in the Boran cattle used in the ECF infection kinetics study at ILRI in 1981 (Morrison, et al., 1981).

From day 8–11 post infection, total leukocyte counts dropped significantly, with nadirs of 300–3000 leukocytes/μL of blood (normal 4,000–12,000/μL). Both neutrophil and lymphocyte subsets were affected; however, neutropenia was far more severe than lymphopenia in all animals. The Boran cattle infected at ILRI in 1981 also developed severe terminal leukopenia [31]. In our study, four calves developed severe thrombocytopenia, with nadirs of 42,000–59,000 platelets/μL of blood (normal 100,000–700,000/μL). The development of thrombocytopenia corresponded to the development of mucosal petechiation. Terminally, three calves (1415, 1419, and 1435) developed mild anemia (PCV 21%-23%, pre-infection PCV for these calves was 29%-31%), and all animals developed moderate hypoproteinemia. Beginning seven days post infection, schizont-infected lymphoblasts were consistently detected on Giemsa-stained blood smears; however, merozoite infected erythrocytes were rarely detected. Sustained, moderate liver enzyme elevations were noted in two of five (1415 and 1412) animals from day seven post infection onward.

### Gross Lesions of ECF in Holstein calves

Necropsy findings were similar in all animals. The pleural, pericardial, and peritoneal cavities contained abundant free serosanguinous fluid (S1 Fig), and petechiae and ecchymoses were disseminated across the epicardium, tracheal mucosa, visceral pleura, and serosa of the forestomachs, and intestines. All animals had severe interstitial pneumonia affecting all lung lobes, as well as severe pulmonary edema, with expansion of interlobular septae and abundant foam within the airways (S2 Fig). Bilaterally, the retropharyngeal, parotid, cervical, prescapular, tracheobronchial, pre-hepatic and sublumbar lymph nodes were enlarged to 2–20 times normal size. On cut surface, enlarged nodes were edematous, and contained large foci of hemorrhage and necrosis. Two animals (1415 and 1420) had severe, subcutaneous intermandibular edema. Calf 1415 also had severe fibrinosuppurative tracheitis and bronchopneumonia (secondary bacterial infection).

### Histopathologic lesions of ECF in Boran and Holstein calves

**Fig 1:**
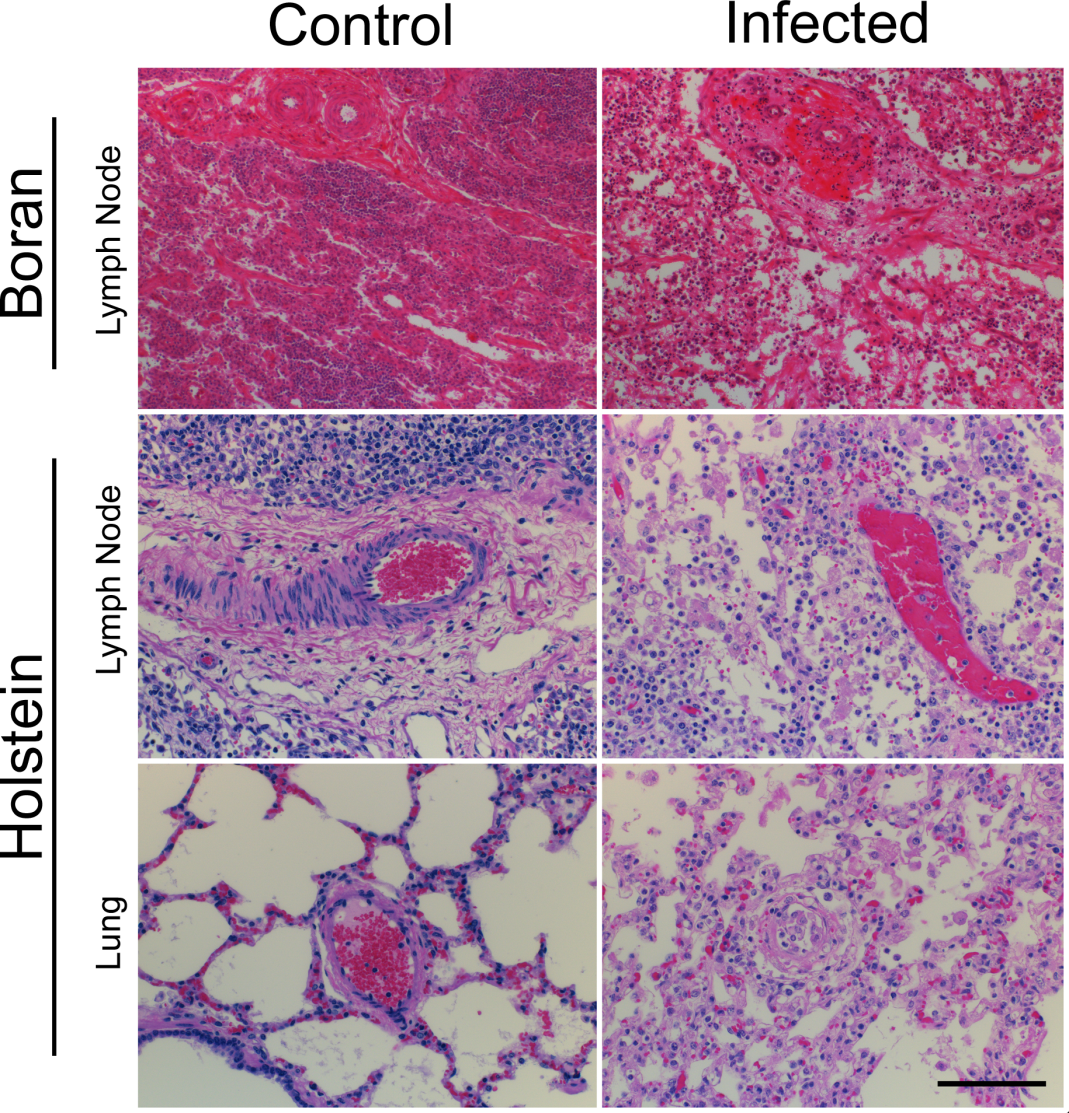
Histologic Lesions in Holstein and Boran cattle. Representative photomicrographs of H&E stained sections of lymph node medulla in uninfected control and infected Boran (day 12) and Holstein calves, and lung from uninfected control and infected Holstein calves. Note: Severe disruption of vessel walls by mononuclear cells and fibrinoid degeneration (lung and lymph node), and lymphohistiocytic interstitial pneumonia with edema (lung). Scale bar: 200 μm.

In order to provide a more comprehensive understanding of ECF pathogenesis, histologic evaluation of the lungs, lymph nodes, spleen, liver, heart, brain, and intestines of Holstein calves with terminal ECF was performed. In multiple foci throughout the lungs of all calves, small to medium caliber blood and lymphatic vessel walls were severely disrupted by fibrinoid degeneration, necrotic cellular debris, edema, and macrophages and lymphocytes (mononuclear vasculitis). Affected vessels were lined by reactive endothelial cells, and clusters of monocytes were frequently adhered to the luminal aspect of the endothelium. In general, thin-walled veins and lymphatic vessels were more severely affected than arterioles. Alveoli and interlobular septae were filled with and widened by edema, fibrin, hemorrhage, and alveolar macrophages, and alveolar septae were markedly widened by fibrin mats and infiltrating macrophages and lymphocytes (Fig 1).

Within lymph nodes, corticomedullary distinction was largely lost, and normal architecture replaced by sheets of macrophages and lymphoblasts separated by large aggregates of necrotic cells, fibrin and hemorrhage. As in the lungs, small to medium caliber blood and lymphatic vessels within lymph nodes were often disrupted by vasculitis. Lymph nodes draining the site of infection were most severely affected (Fig 1).

Hepatic portal triads were rimmed by moderate to large numbers of macrophages and lymphoblasts, and portal vessels were multifocally, mildly disrupted by vasculitis.

Within the spleen, the distinction between the red and white pulp was often unapparent. In some foci, periarteriolar lymphatic sheaths were often greatly expanded by monomorphic populations of lymphoblasts or replaced by sheets of macrophages. Numerous macrophages were filled with hemosiderin, suggesting increased erythrocyte turnover and erythrophagocytosis.

In order to establish a kinetic framework for the development of histologic lesions in ECF, sections of lymph nodes collected from Boran cattle at regular intervals following *T*. *parva* infection were examined and compared. Within these sections, mild vasculitic lesions were evident as early as three days post infection. From day 10 to 19, severe lymphoid necrosis, edema and hemorrhage developed within affected nodes. During this time period, vasculitis lesions became more prevalent and more severe, with marked fibrinoid degeneration, endothelial disruption, and fibrin thrombus formation observed in the majority of vessels by day 19. Representative histologic lesions are shown in Fig 1.

### Tissue distribution of T. parva infected cells

**Fig 2:**
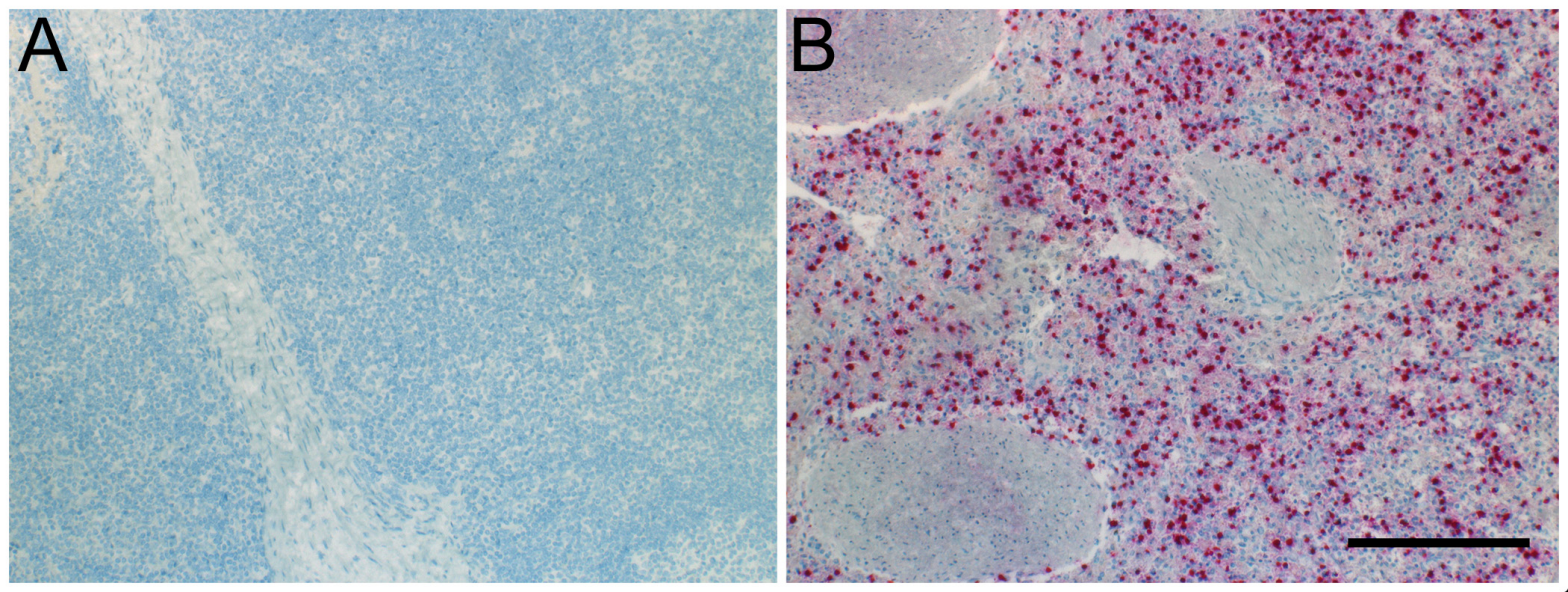
Splenic PIM Immunoreactivity. Shown are the results of immunohistochemical labeling of the *T*. *parva* antigen, PIM, in the spleen. **A**. The spleen of the control calf lacks PIM immunoreactivity. **B.** In a representative infected Holstein calf, there is abundant PIM immunoreactivity within lymphocytes of the red and white pulp. Scale bar: 200 μm.

In all infected animals, large numbers of schizont-infected cells were detected within the lungs, lymph nodes, spleen, liver, and bone marrow, and rare infected cells were detected within the brain, adrenal glands, thyroid gland, heart, and tongue. Schizont-infected cells were most prevalent in the spleen (Fig 2), and were widely dispersed throughout the red and white pulp. Within the lungs, infected cells infiltrated interlobular and alveolar septae, and were occasionally within perivascular connective tissue and vessel walls. Infected cells were within the cortex and medulla of lymph nodes, and often surrounded portal vessels within the liver. No staining with the anti-schizont antibody was detected in tissues from an uninfected control calf.

### Immunohistochemical characterization of mononuclear cell tissue infiltrates

**Fig 3:**
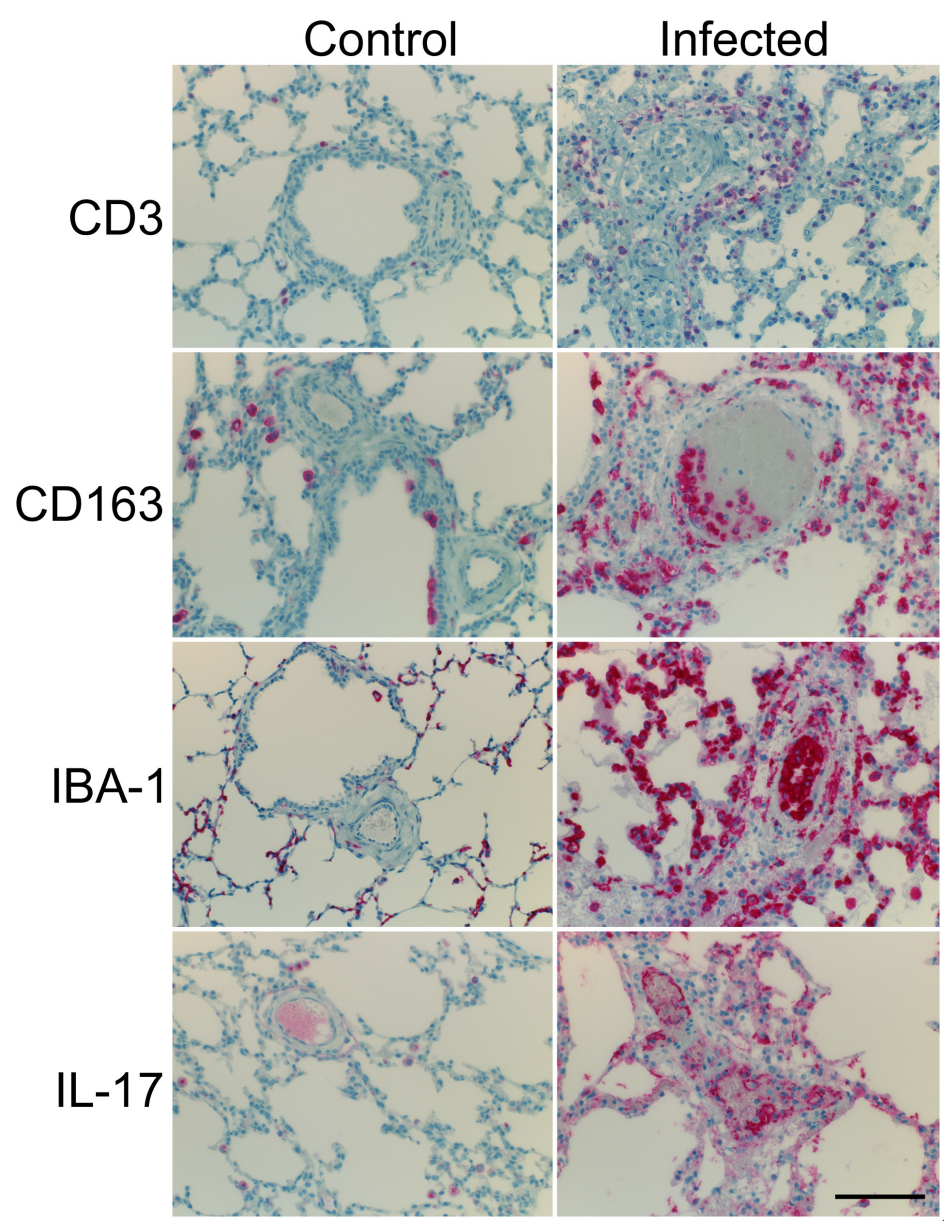
Immunohistochemical Labeling of Leukocytes in the Lungs. Shown are the results of IHC labeling for CD3-like immunoreactivity (CD3-li), CD163-li, IBA-1-li, and IL-17-li in the lung of uninfected control and representative infected Holstein calves. Note: CD3-li, CD163-li, and IL-17-li is significantly increased in the lungs of the infected calf. Furthermore, CD3+, CD163+, IBA-1+ and IL-17+ mononuclear cells are components of vasculitis lesions. Vascular endothelial cells consistently exhibit pronounced IL-17-li in the infected calf, but endothelial IL-17-li is rare in the control calf. Scale bar: 200 μm.

In all animals, the lungs, lymph nodes, spleen, and liver contained markedly increased numbers of T cells (CD3+ cells); however, B cells (CD20+ cells) were rarely detected within the lungs and liver, and were greatly decreased within the spleen and lymph nodes (S3 Fig). Within the lungs, T cells were found within interlobular and alveolar septae, within perivascular connective tissue, and within vessel walls and lumina (Fig 3). Large sheets of blastic T cells expanded the red and white pulp of the spleen and the cortex and medulla of lymph nodes, and were often detected within vessel walls of both organs. T-cells expanded the periportal space within the liver.

The lungs, lymph nodes, spleen, and liver of all infected animals contained large numbers of IBA-1 positive (histiocytic) cells. Within the lungs of infected animals, large numbers of macrophages infiltrated vessel walls, alveolar septae, alveoli, and interlobular septae, and large clusters of macrophages were often adhered to the endothelium of small to medium caliber vessels. In lung sections from the control animals, normal alveolar macrophages were observed in the alveolar septae. In the lymph nodes of infected calves, large sheets of macrophages obscured normal corticomedullary architecture, disrupted vessel walls, and partially occluded vessels. In lymph nodes from the infected calf, macrophages were confined to the subcapsular and medullary sinuses. Within infected animals, but not uninfected controls, sheets of macrophages multifocally disrupted the red and white pulp of the spleen, and small to moderate numbers of macrophages were mixed with lymphocytes in periportal areas of the liver. Further sub-typing of macrophage populations using CD163 labeling revealed that the vast majority of tissue-associated macrophages within infected animals were CD163+ (Fig 3). The mean percent sectional area positive for CD163 and IBA-1 in lung sections from *T*. *parva* infected and control calves is listed in Table 3. The mean percent sectional area positive for CD163 was significantly greater in the *T*. *parva* infected calves than in the control calves (p = 0.005). This finding supports the histologic impression that the lungs of infected calves contain larger numbers of CD163+ macrophages than those of the control calves. Although the CD163+ macrophage subset is increased in the lung during acute ECF, there was no significant difference in total histiocytic cell (IBA-1 positive) sectional area in *T*. *parva* infected animals and control animals (p = 0.28).

**Table 3:** Mean Sectional Area Positive for CD163, IBA-1, and IL-17.

Marker	Mean Sectional Area Positive		p Value
	Infected (n = 4)	Control (n = 3)	
CD163	23.02%	8.97%	0.005
IBA-1	26.28%	22.61%	0.28
IL-17	24.77%	7.5%	0.01

### Expression of IL-17 by macrophages

In all infected animals, the lungs, lymph nodes, spleen, and liver contained large numbers of IL-17-positive mononuclear leukocytes. Within the lungs, IL-17-positive cells were found within alveolar and interlobular septae, alveoli, and within vessel walls and vasculitic lesions (Fig 3). Sheets of IL-17-positive cells were most abundant within the subcapsular and medullary sinuses of lymph nodes, and small numbers of IL-17-positive cells were found within the superficial cortex. IL-17-positive cells were often found within periportal regions of the liver, and scattered throughout sheets of macrophages within the spleen. Importantly, vascular endothelial cells were frequently strongly positive for IL-17 (Fig 3). Non-cell associated IL-17 staining was frequently observed within mats of fibrin and within connective tissue of vessel walls. The mean percent sectional area positive for IL-17 in lung sections from *T*. *parva* infected and control calves is listed in Table 3. The mean sectional area positive for IL-17 within the lungs of *T*. *parva* infected cattle was significantly greater than that in the control cattle (p = 0.01).

**Fig 4:**
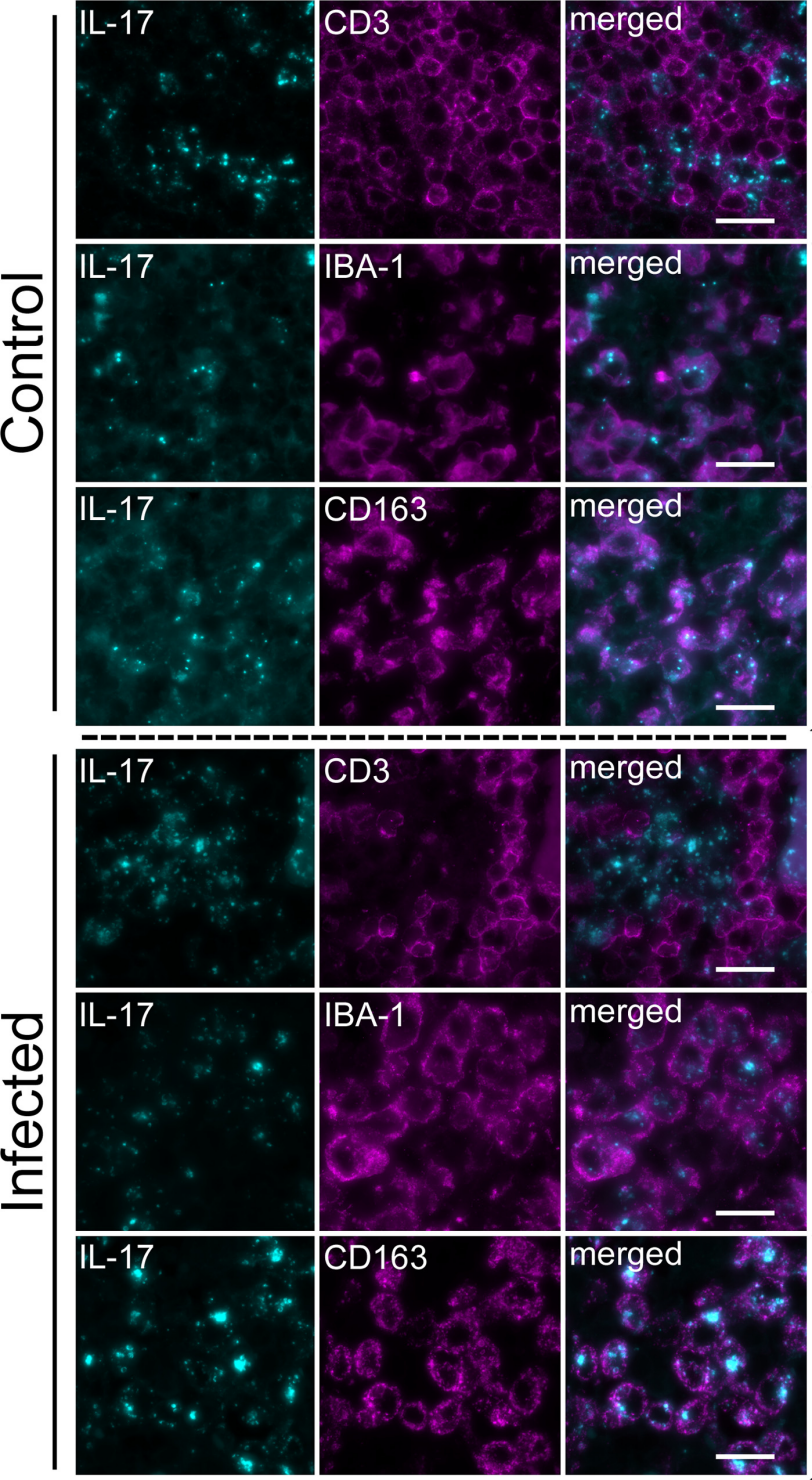
Dual Fluorescence Labeling of Leukocytes in Lymph Nodes. Shown are the results of dual fluorescence labeling for IL-17-li (pseudocolored cyan) and either CD3-, IBA-1-, or CD163-li (pseudocolored magenta) in the medulla of a lymph node from a control calf and representative infected calves. Note: IL-17-li was infrequent in the medulla of control calf lymph node but was widespread throughout the medulla of infected calves. Where it occurred within the non-vascular elements, IL-17-li was generally punctate and appeared intracellular. The cell-associated, punctate IL-17-li was considerably weaker in intensity and less dense as compared to the infected calves. In both the infected calves and, where present, in the control calf, intracellular punctate IL-17-li was most frequently co-localized with IBA-1-li cells and CD163-li cells but not with CD3-li cells. Scale bar: 20 μm.

**Fig 5:**
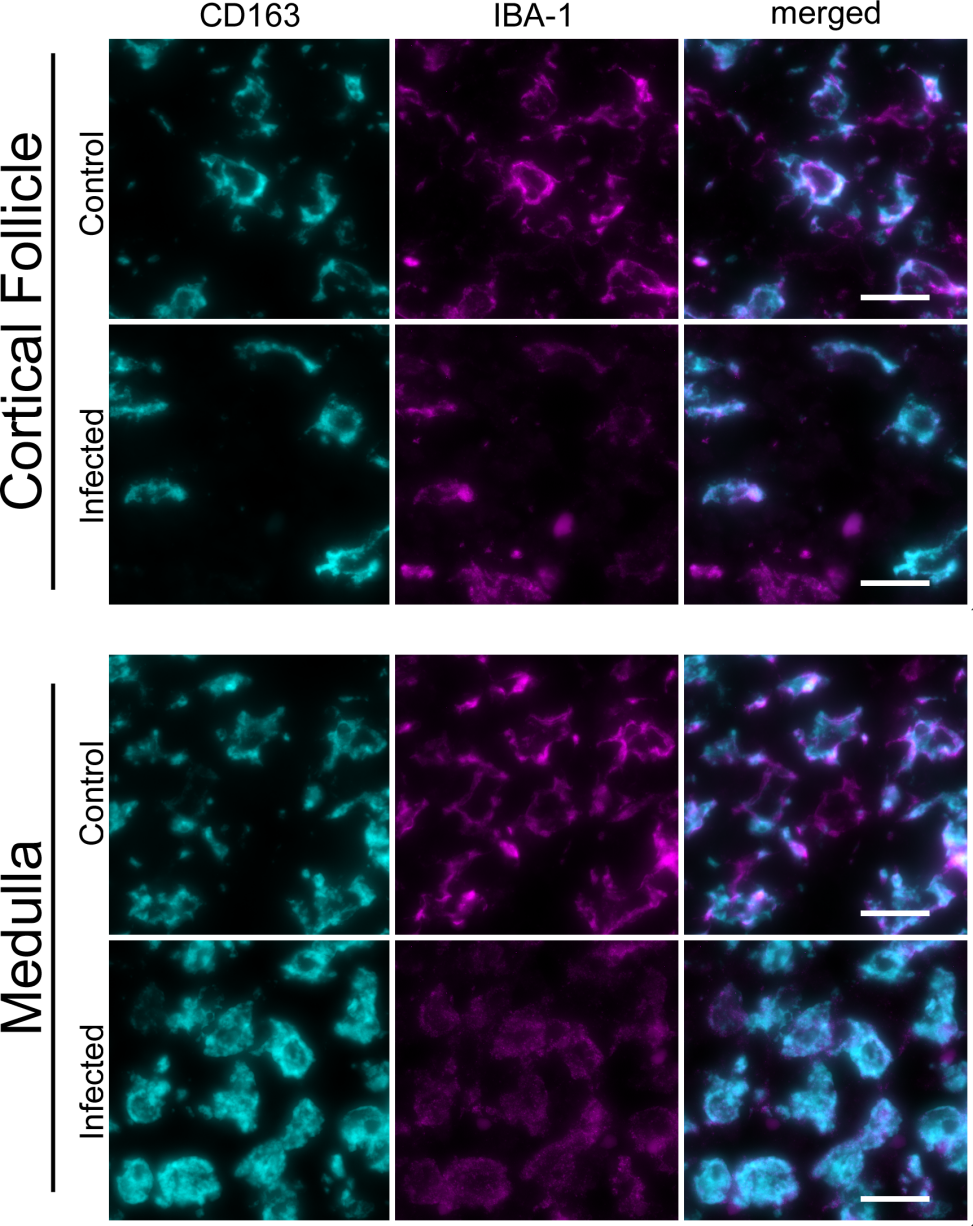
CD163 and IBA-1 Dual Fluorescence Labeling. Shown are the results of dual fluorescence labeling for CD163-li (pseudocolored cyan) and IBA-1-li (pseudocolored magenta) in a lymph node cortical follicle and medulla from the control calf and representative infected calves. In both calves, most CD163-li cells were co-labeled by IBA-1-li but not all IBA-li cells were co-labeled with CD163-li. IBA-1-li cells without definitive CD163-li were most frequent in the control calf lymph parafollicular regions. Scale bar: 20 μm.

Within the lymph nodes and lungs of both the infected and control calves, almost all IL-17-positive mononuclear cells were co-positive for IBA-1, and only rarely co-positive for CD3, suggesting that, in acute *T*. *parva* infection, IL-17 is produced predominantly by macrophages (Fig 4). Interestingly, in all calves, the majority of IL-17-positive macrophages co-expressed CD163 (Fig 4). The number of IL-17-positive cells was significantly greater in the infected calves than in the control cattle. Since CD163 is only expressed on a subset of macrophages, and is up-regulated in actively phagocytic cells, we performed co-labeling studies using IBA-1 and CD163. As expected, all CD163-positive cells were also positive for IBA-1, but only a subset of IBA-1 positive cells were also positive for CD163 (Fig 5). In the infected cattle, a subjectively larger proportion of IBA-1-positive (histiocytic) cells co-expressed CD163 than in the control cattle.

## Discussion

The results of this study provide new insights into the immunopathogenesis of acute, fatal ECF. Protozoa—including *Plasmodium* sp., *Trypanosoma* sp., and *Toxoplasma* sp., are adept at immune system subversion, and numerous pathogen-mediated forms of immune evasion—such as antigenic variation, limited antigen production, persistence in immune-privileged sites, and altered antigen presentation have been described in protozoal infections [32–35]. Protozoa have also been shown to subvert T-cell responses by triggering anergy, exhaustion, or apoptosis [36]. Another mechanism of T-cell inhibition in protozoal disease is the induction of alternatively activated, or suppressor, macrophages [37]. Although definitive phenotypic characterization of macrophage subsets *in situ* is difficult because gene expression in macrophages is highly plastic and changes with fluctuating environmental cues [38], alternatively activated macrophages in humans and mice are often characterized by high expression of CD163 [37].

CD163 is a membrane-bound, hemoglobin/haptoglobin scavenger receptor protein in the scavenger receptor cysteine-rich superfamily group B [39]. Hemoglobin is released from damaged erythrocytes, and can cause oxidative damage to cells. After binding to CD163, hemoglobin is degraded within the macrophage, and several anti-inflammatory substances are released [40, 41]. In addition to its scavenger receptor role, CD163 can also bind bacteria and viruses, and its expression is up-regulated on mature, actively phagocytic tissue macrophages [19]. In general, CD163 expression is up-regulated by glucocorticoids and IL-10, and down-regulated by pro-inflammatory cytokines, including IFN-γ [20].

In infectious disease, alternatively activated macrophages have been shown to suppress T-cell activation. The presence of CD163^+^ macrophages is associated with increased tissue invasion, decreased T-cell activity, and resultant poor prognosis in many forms of human cancer [42, 43], and alternatively activated macrophages are associated with altered T-cell function and parasite persistence in trypanosomiasis [44–46] and toxoplasmosis [47]. It is possible that lysis of *T*. *parva* infected lymphocytes and erythrocytes, and the resulting free iron and pro-inflammatory milieu of necrosis stimulates a robust CD163+ macrophage response in acute *T*. *parva*. The presence of large numbers of CD163+ macrophages, could, in turn, inhibit necessary proliferative and cytotoxic T-cell responses. Alternatively, since *T*. *parva* infected lymphocytes have been shown to produce significant amounts of IL-10 [48], it is possible that schizont-infected lymphocytes themselves stimulate a CD163 macrophage response in acute ECF.

Robust CD163+ macrophage responses are also observed in macrophage activation syndrome (MAS, also referred to as hemophagocytic lymphohistioctyosis), an exaggerated systemic macrophage response observed in numerous neoplastic, autoimmune, and infectious diseases [49], including trypanosomiasis in cattle [50]. Clinical components of MAS include prolonged fever, lymphadenomegaly, splenomegaly, hemorrhage, cytopenias that affect at least two leukocyte lineages, thrombocytopenia, hypoproteinemia, elevated liver enzymes, hemophagocytosis, tissue infiltration by activated macrophages, hyperferritinemia, and elevated soluble CD163 (sCD163) [51]. CD163 is cleaved from the membrane of macrophages in response to severe inflammation, exposure to LPS, and cross linkage of FC receptors [19]. In humans and mice, serum levels of sCD163 increase markedly during acute inflammation, and serve as a means of monitoring macrophage activation [52, 53].

The cattle in this study demonstrated several of the hallmark criteria of MAS, including fever, lymphadenomegaly, hemorrhage, panleukopenia, thrombocytopenia, hypoproteinemia, elevated liver enzymes, and infiltration of numerous tissues by macrophages. Consistent with MAS, the spleens of these cattle contained increased numbers of macrophages, the majority of which were CD163+. Many macrophages contained hemosiderin pigment, which is consistent with hemophagocytosis and increased erythrocyte turnover. Large numbers of schizont-infected lymphocytes were present in the spleens of the animals. Elucidation of the direct effect of schizont-infected lymphocytes on macrophage induction in acute ECF requires further study. As assays do not yet exist to quantify soluble CD163 in the serum of cattle, we were unable to measure sCD163 in this study.

Interestingly, many of the CD163+ macrophages also exhibited positive immunolabeling for IL-17, a pro-inflammatory cytokine. CD163 is considered a multifunctional receptor, and thus, CD163+ macrophages often serve multiple, disparate functions [19]. Monoclonal antibody binding to CD163 caused production of pro-inflammatory cytokines in rats [54], and engagement of CD163 by bacteria also led to pro-inflammatory cytokine production [55]. Thus, CD163+ cells may play myriad immunomodulatory roles throughout the disease course in ECF.

It is likely that the marked macrophage-mediated IL-17 response contributes significantly to the development of severe lesions in fatal ECF. In many protozoal infections, including *Leishmania major* [21], *Toxoplasma gondii* [23] and *Eimeria tenella* [22], severe tissue damage is associated with an overly robust IL-17 response, and can often be abrogated by administration of anti-IL-17 antibodies [22].

IL-17 can be produced by many leukocyte subsets, including Th17 CD4+ cells, CD8+ T cells, γδ T cells, macrophages, dendritic cells and NK cells [56], and acts on many cell types, including leukocytes, endothelial cells, epithelial cells, chondrocytes, osteoclasts, and fibroblasts. Binding of IL-17 to the IL-17 receptor leads to activation of MAP-kinase, NF-κB, and C/EBPβ pathways, leading to production of numerous chemokines, cell adhesion molecules, matrix metalloproteinases, and antimicrobial substances [57]. In psoriasis, activation of ERK and MAP kinases after IL-17 binding to endothelial cells leads to increased cell adhesion molecule and chemokine expression and vascular inflammation [58]. IL-17 production is also a component of giant cell arteritis, and is believed to exert its effects on endothelial cells and fibroblasts within vessels, leading to endothelial cell activation, increased expression of adhesion molecules and cytokines, recruitment of mononuclear cells, and vascular damage [59, 60]. In this study, endothelial cells were strongly positive for IL-17, suggesting that IL-17 produced by leukocytes in the affected tissues was binding to endothelial cells. Downstream effects of IL-17 binding to endothelial cells likely gave rise to the severe vasculitis noted in many tissues in these calves.

Significantly, the most severe vasculitic lesions were present within the lungs of both Boran and Holstein calves, suggesting that the pathogenesis of ECF is the same in both *Bos indicus* and *Bos taurus* cattle. Inflammation of pulmonary blood and lymphatic vessels is the cause of terminal pulmonary edema in ECF. In this, vasculitis leads to leakage of protein-rich fluid into the airways and pleural space, causing severe restrictive and obstructive respiratory distress, hypoxia, and death. The histological and immunohistochemical findings of this study further suggest *Theileria parva* infection triggers macrophage activation syndrome and IL-17 production leading to the development of vasculitis.

Future work will be undertaken to determine the cellular mechanisms of macrophage activation and the effect of CD163+ macrophages on T-cell function. This enhanced understanding of the role of the innate immune response in *T*. *parva*, and its subsequent effect on the trajectory of the adaptive immune response, will aid in the development of a vaccine that elicits an efficacious cytotoxic T-cell response without causing severe immunopathology. Furthermore, the results of this study suggest that immunosuppressive drug therapy or anti-IL-17 therapy may provide clinical benefit to cattle with ECF.

## Supporting Information

S1 Fig Severe Pleural and Peritoneal Effusion, Calf 1435. In all deceased calves, large amounts of free pleural and peritoneal fluid was noted during the gross exam.
(TIF)

S2 Fig Severe Interstitial Pneumonia and Pulmonary Edema, Calf 1435. In all deceased calves, lungs were reddened, wet, and heavy, and there was marked expansion of interlobular septae by edema.
(TIF)

S3 Fig CD20 Immunohistochemical Labeling in the Lungs. In all cattle, CD20-positive cells (B lymphocytes) were rare in the lungs. Depicted is part of a normal bronchiolar associated lymphoid follicle. Note the lack of B lymphocytes within alveolar septae, vessel walls, and vessel lumina. Scale bar = 200 μm.
(TIF)

S1 Table Raw Morphometric Data, IL-17. (XLSX)

S2 Table Raw Morphometric Data, IBA-1. (XLSX)

S3 Table Raw Morphometric Data, CD163. (XLSX)
