# Supplementary figures and images for: East Coast Fever Caused by Theileria parva Is Characterized by Macrophage Activation Associated with Vasculitis and Respiratory Failure
Source: PLoS One. 2016 May 19;11(5):e0156004. doi: 10.1371/journal.pone.0156004 (PMC4873194; doi:10.1371/journal.pone.0156004)

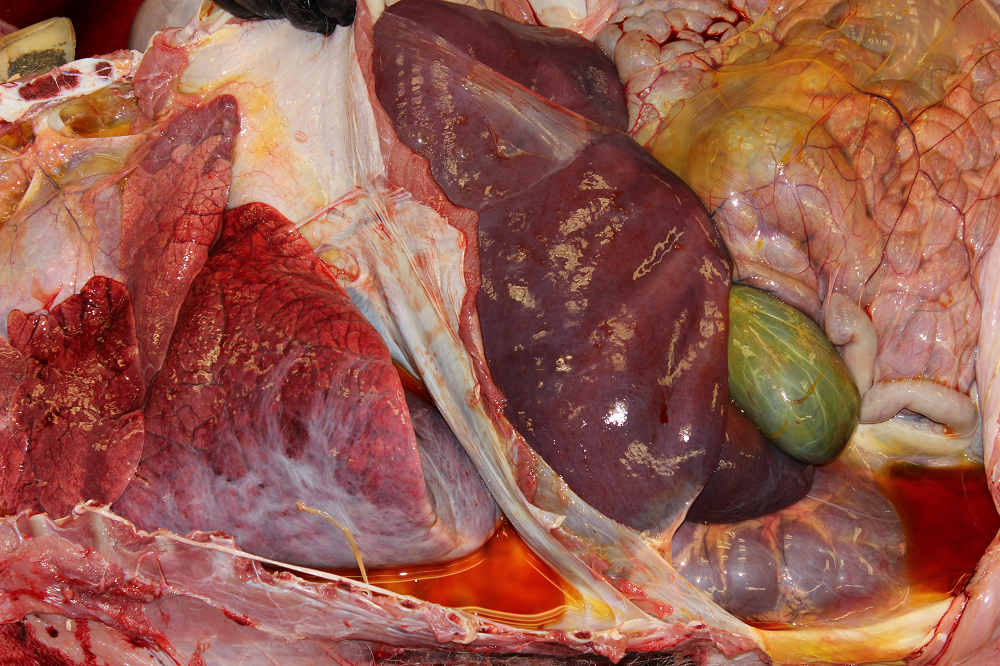

Supplement: S1 Fig — In all deceased calves, large amounts of free pleural and peritoneal fluid was noted during the gross exam. (TIF) [file pone.0156004.s001.tif]

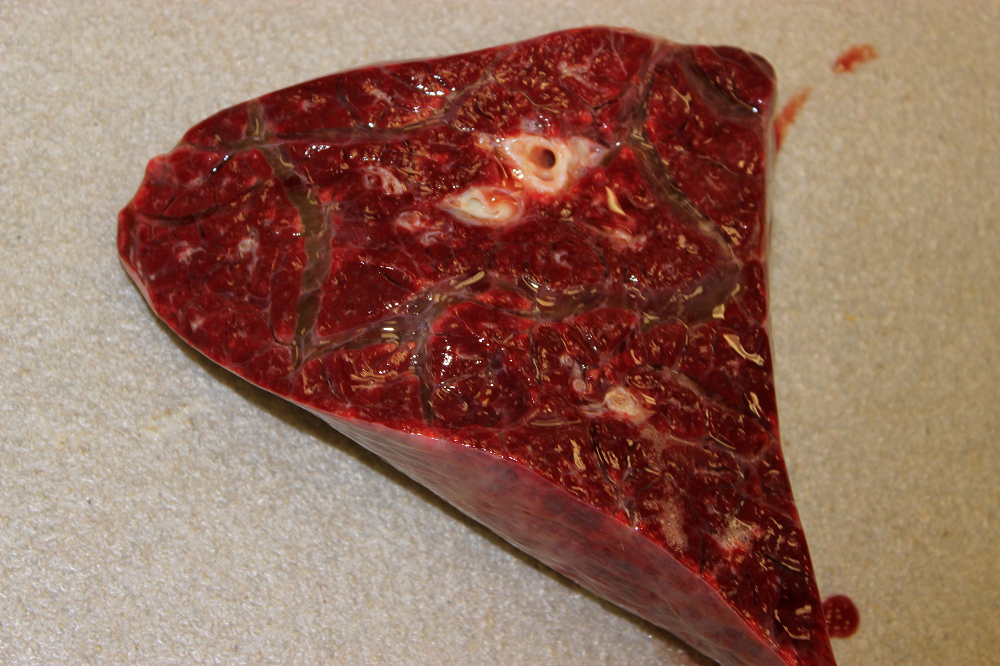

Supplement: S2 Fig — In all deceased calves, lungs were reddened, wet, and heavy, and there was marked expansion of interlobular septae by edema. (TIF) [file pone.0156004.s002.tif]

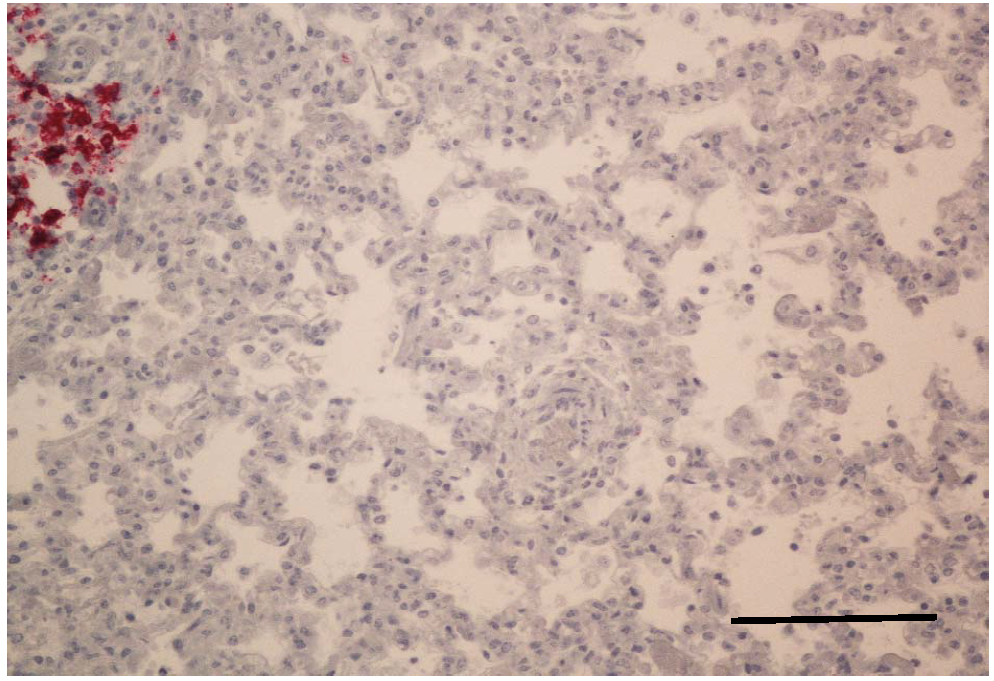

Supplement: S3 Fig — In all cattle, CD20-positive cells (B lymphocytes) were rare in the lungs. Depicted is part of a normal bronchiolar associated lymphoid follicle. Note the lack of B lymphocytes within alveolar septae, vessel walls, and vessel lumina. Scale bar = 200 μm. (TIF) [file pone.0156004.s003.tif]
